# Cerebello‐Basal Ganglia Functional Connectivity Differences in Alzheimer’s Disease and Mild Cognitive Impairment

**DOI:** 10.1002/alz.095763

**Published:** 2025-01-09

**Authors:** Ivan Herrejon Gonzales, T. Bryan Jackson, Tracey Hicks, Jessica A Bernard

**Affiliations:** ^1^ Texas A&M University, College Station, TX USA; ^2^ Vanderbilt Memory & Alzheimer’s Center, Vanderbilt University Medical Center, Nashville, TN USA

## Abstract

**Background:**

Extensive research on the cerebral cortex and the hippocampus has improved our understanding of mild cognitive impairment (MCI) and Alzheimer’s disease (AD). Historically, however, the cerebellum’s role in these stages of cognitive decline has been traditionally neglected due to its associations with motor function. Recent research has demonstrated cerebellar structural and connectivity differences in MCI and AD. Further investigating is however needed. In cognitively normal (CN) older adults cerebello‐basal ganglia (CB‐BG) functional connectivity is lower compared to younger adults. However, it remains an open question how these networks differ across different stages of cognitive decline and whether they are associated with cognitive and/or task performance.

**Method:**

Here, we investigated CB‐BG resting state networks associated with motor and cognitive cortical circuits across CN, MCI, and AD populations. The Alzheimer’s Disease Neuroimaging Initiative (ADNI‐3) was used to obtain the data of 478 participants who completed a motor and cognitive battery. All participants also underwent resting state functional magnetic resonance imaging (fcMRI). Analysis was completed using the CONN toolbox.

**Result:**

We found a higher FC between Crus I with globus pallidus *pars externa* and lower FC between lobule VIIIb and dorsal caudal putamen in AD compared to CN individuals. However, no significant results were found when comparing AD to MCI or MCI to CN, suggesting that this stage of cognitive decline does not affect cerebello‐basal ganglia networks

**Conclusion:**

Further understanding of network dysfunction that includes the cerebellum can help inform future clinical work and elucidate the mechanisms of MCI and AD.

